# Self-Esteem in Adults With ADHD Using the Rosenberg Self-Esteem Scale: A Systematic Review

**DOI:** 10.1177/10870547241237245

**Published:** 2024-03-16

**Authors:** Aksel Bjørø Pedersen, Bernhard Vestby Edvardsen, Salvatore Matias Messina, Maria Rudjord Volden, Lisa L. Weyandt, Astri J. Lundervold

**Affiliations:** 1 Department of Biological and Medical Psychology, University of Bergen, Bergen, Norway; 2 Department of Psychology, University of Rhode Island, Kingston, USA

**Keywords:** systematic review, self-esteem, Rosenberg self-esteem scale, ADHD, adults

## Abstract

**Objective:**

To summarize and analyze recent articles investigating self-esteem in adults with ADHD, focusing on the impact of demographic and clinical characteristics, and methodological issues.

**Method:**

Following PRISMA guidelines, a systematic search for literature published between 2010 and 2022 was conducted in the Web of Science, Ovid, Pubmed, and EBSCO databases.

**Results:**

Eleven studies met inclusion criteria. Five of the six studies including healthy controls reported lower self-esteem in participants with ADHD. ADHD symptoms correlated negatively with self-esteem. Gender differences were not observed. Self-esteem mediated several outcomes associated with ADHD. There was a lack of studies that examined potential mechanisms behind the reduced self-esteem, and studies controlling for confounding variables.

**Conclusion:**

A robust association between ADHD and low self-esteem in adults emerged, but the lack of control of confounding variables is critical to consider when interpreting the findings. Longitudinal studies addressing the limitations of the current studies are needed.

## Introduction

ADHD is a neurodevelopmental disorder with core symptoms of inattention, hyperactivity, and impulsivity (American Psychiatric Association [APA], 2013). ADHD is characterized by three different presentations; predominantly inattentive, predominantly hyperactive/impulsive, and combined. The worldwide prevalence rate of ADHD in childhood is reported to be between 5% to 7% (Polanczyk et al., 2007; Thomas et al., 2015). At least two-thirds of childhood cases are reported to show persistent symptoms of the disorder into adulthood (Faraone et al., 2006), and the estimated worldwide prevalence of ADHD in adulthood is approximately 2,5% (Kooij et al., 2016; Simon et al., 2009; Song et al., 2021). Boys are diagnosed with ADHD more frequently than girls. However, the gender distribution in adulthood is more balanced, with inattention being the most prevalent presentation (Hinshaw et al., 2022; Willcutt, 2012). A higher prevalence of ADHD is reported in the white American population compared to the African American, Hispanic, and Asian American population (Chung et al., 2019).

ADHD in adulthood is associated with a wide array of challenges in everyday life, including problems affecting social and emotional function (Kooij et al., 2019; Paulson et al., 2005), emotional regulation (Groves et al., 2022), educational attainment (Gjervan et al., 2016; Henning et al., 2022), employment level (Gjervan et al., 2016), quality of life (Kazda et al., 2022; Pinho et al., 2019), and the presence of chronic fatigue (Kooij et al., 2019), somatic diseases (Instanes et al., 2018), neuropsychological deficits (Halleland et al., 2012; Lundervold et al., 2019; Munro et al., 2018; Weyandt et al., 2017), and psychiatric disorders (Franke et al., 2018; Kooij et al., 2019; Nutt et al., 2007; Schiweck et al., 2021; Torgersen et al., 2006). The most common co-occurring psychiatric disorders in adult ADHD are depression, anxiety disorders, bipolar disorder, substance use disorders, and personality disorders (Katzman et al., 2017). Beyond these symptoms and challenges, ADHD is associated with impaired self-esteem in both childhood and adulthood (Çelebi & Ünal, 2021; Cook et al., 2014; Kooij et al., 2010). The interplay between ADHD and self-esteem is complex, and stigma toward individuals with ADHD may add to the burden of the disorder (Halleröd et al., 2015). While empirical evidence regarding casual impacts on self-esteem in ADHD may be scarce, it has been theoretically suggested that the negative life experiences and failures associated with ADHD, and the consecutive negative feedback, may have detrimental effects on self-esteem (Young & Bramham, 2006). Recognizing challenges and stigma faced by adults with ADHD may therefore be an important first step to help promote a more positive self-esteem in these individuals.

Self-esteem is a core construct in the psychological literature that is theoretically and empirically associated with life quality and psychological well-being (Kashdan, 2004; Robins et al., 2001). Morris Rosenberg (1979, p. 23) describes the self as “one of the objects toward which one has . . . feelings,” and states that self-esteem “refers to a positive or negative evaluation of the self” (Rosenberg, 1979, p. 31). Low self-esteem is thought to manifest as negative attitudes toward the self, with low self-acceptance and self-respect, and feelings of low self-worth (Rosenberg, 1979; Rosenberg & Simmons, 1972). Rosenberg developed the Rosenberg Self-Esteem Scale (RSES) (1965). According to Donnellan et al. (2015), RSES is the most frequently used questionnaire to assess self-reported self-esteem, accounting for more than 50% of the citations of studies using self-esteem measures. The tool was originally developed using a Guttman scale, with four options for each item (Rosenberg, 1965). RSES consists of ten items, where five are formulated positively (e.g., “I feel that I am a person of worth, at least on an equal plane with others.”) and five negatively (e.g., “All in all, I am inclined to feel that I am a failure.”). Due to the complexity of the Guttman scale, the RSES is often modified to a Likert scale of 4 points (Jordan, 2020), and a score below 50% has been suggested as a cutoff for low self-esteem (Isomaa et al., 2013). RSES has shown high cross-cultural validity (Schmitt & Allik, 2005).

Numerous important factors have been associated with the development of self-esteem. Age seems to be one critical factor, based on meta-analytic findings of the developmental trajectory of self-esteem (Orth et al., 2018; Orth & Robins, 2014). In the general population, self-esteem has been found to steadily increase from childhood through adolescence and adulthood, peaking at 50 or 60 years of age. After peaking, self-esteem has been found to decline, with a rapid decline starting around age 70 years. These differences can be attributed to varying challenges, cognitive, emotional, and social. A recent meta-analysis (Betancourt et al., 2024) suggests that children and adolescents with ADHD have reduced global, academic, and social self-esteem compared to those without ADHD. As children with ADHD progresses to adolescence and adulthood, the comparison to peers and demands from society increases, potentially inflicting harm to their self-esteem (Richman et al., 2010, p. 112). Increased academic and social demands could elucidate findings suggesting that the inattentive presentation of ADHD appears to have the most devastating effect on self-esteem in adolescents (Kita & Inoue, 2017). Throughout adulthood, males are shown to display slightly higher self-esteem compared to females (Orth et al., 2010), but gender does not appear to significantly impact the developmental trajectory of self-esteem (Orth et al., 2018). Meta-analyses have found differences in levels of self-esteem between different ethnic populations, with African Americans scoring higher than white Americans, and Asian Americans scoring even lower (Twenge & Crocker, 2002). Others have found associations between self-esteem and education level (Orth et al., 2010, 2012; Twenge & Campbell 2002; von Soest et al., 2018), employment status (Kuster et al., 2013; Orth et al., 2012; von Soest et al., 2018), and job satisfaction (Kuster et al., 2013; Orth et al., 2012). These studies were based on longitudinal prospective designs, where in most instances no reciprocal effects of these outcomes on self-esteem were detected. Low self-esteem is also associated with various psychiatric disorders such as depression, anxiety, premorbid psychosis, borderline and avoidant personality disorders, and eating disorders (Bemrose et al., 2021; Colmsee et al., 2021; Lynum et al., 2008; Šare et al., 2021; Sowislo & Orth, 2013). Furthermore, low self-esteem has been associated with the development of poor physical health (Byth et al., 2022; Trzesniewski et al., 2006).

Knowing that adults with ADHD are at risk for several of the outcomes associated with reduced self-esteem (including lower educational attainment, lower employment level, comorbid psychiatric disorders, and reduced quality of life), the purpose of the present systematic review is to examine the literature from the past decade regarding self-esteem in adults with ADHD with a goal to uncover associated factors potentially impacting self-esteem. Specifically, this study aimed to (a) identify and summarize the peer-reviewed literature analyzing self-esteem in adults with ADHD, and (b) present findings regarding the level of self-esteem and the potential impact of demographic (age, gender, race/ethnicity, education level, and employment status) and clinical characteristics (presentation of ADHD, symptom severity, and comorbid disorders). An additional aim of the systematic review was to identify methodological issues and shed light on crucial gaps in the literature, specifically those raised in a previous review by Cook et al. (2014).

## Methods

This systematic review was conducted according to the Preferred Reporting Items for Systematic Reviews and Meta-Analyses (PRISMA) guidelines (Page et al., 2021). A literature search was conducted in the databases Web of Science, Ovid, Pubmed, and EBSCO over the period 2010 to 2022. The time frame was set with the intention to build upon previous findings based primarily on literature before 2010 (Cook et al., 2014), and thus to ensure relevance to the contemporary context. The search consisted of the following terms: (“ADHD” OR “Attention Deficit Hyperactivity Disorder” OR “hyperactiv*”) AND (“Adult*” OR “Grown up*” OR “Student*”) AND (“self-esteem” OR “RSES” OR “Rosenberg Self Esteem Scale”).

**Figure 1.**
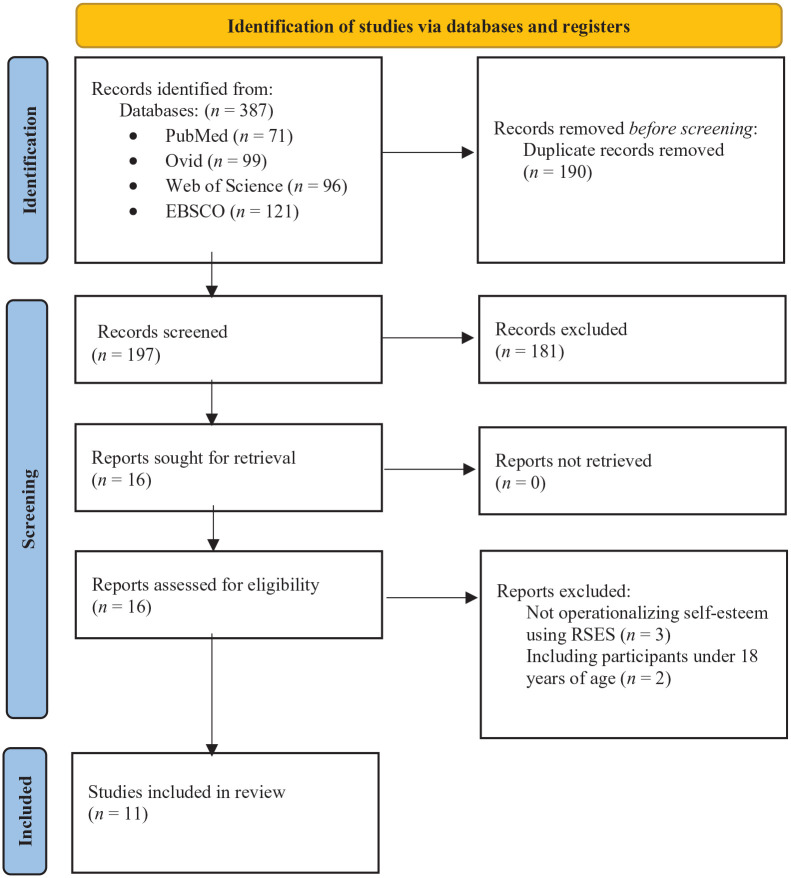
PRISMA 2020 flow diagram (Page et al., 2021).

A flow diagram according to the PRISMA guidelines for the identification of studies is presented in Figure 1. Studies were included if they (1) defined self-esteem by responses on the RSES, (2) reported or analyzed self-esteem in adults with ADHD, (3) operationalized ADHD according to DSM-4 or DSM-5, (4) were peer-reviewed, and (5) published after 2010.

Studies were excluded if they were (1) treatment studies, (2) systematic reviews, (3) book chapters, (4) dissertations, (5) case studies, (6) not written in English, or (7) included participants below the age of 18 years. The first four authors were responsible for the selection process. The authors screened titles and abstracts independently from each other, before reaching a consensus. Further, some studies were excluded based on full-text review.

Only studies using RSES to define self-esteem were included, due to the dominant use of RSES in psychological literature and to maintain consistency between studies. For each study, the following data were retrieved: RSES scores in individuals with ADHD and controls, demographics (age, gender, ethnicity, education, and employment status), symptom severity, presentation of ADHD, and comorbidities. Data were extracted using the data extraction tool developed by Cook et al. (2014). The tool includes the following parameters: description of the subjects, the phenomenon of interest, context, outcome, and study design. The data were extracted independently by the first four authors, and the process was supervised by one of the senior authors (AJL).

To assess the quality and identify possible sources of bias, we applied the quality assessment tool developed by Cook et al. (2014). The quality of each study was expressed in terms of percentage and is presented in Table 1. Questions were rated with yes (score = 2), partially (score = 1), no (score = 0), can’t tell (score = 0), or “not applicable” (item subtracted from the total score). The authors discussed and established a shared understanding of how to employ the assessment tool. Two of the authors independently assessed all articles, and the inter-rater reliability for each study was calculated with Cohens’ Kappa (mean κ = .83, median = 0.87). When a discrepancy in rating occurred, four of the authors met and reached a consensus through discussion.

**Table 1.** Overview of Included Studies.

Author and year	Quality (%)	Sample	Design	ADHD measurement	Key findings
Arsandaux et al. (2021)	72	College students from I-share cohort (France) ADHD symptoms > 18 *n* = 934. ADHD symptoms 14 to 17 *n* = 919. ADHD symptoms <14 *n* = 478. (*M*_age_ total = 20.7, 79.5% female)	Longitudinal cohort study—quantitative design (prospective)	ASRS 1.1	Symptom severity of ADHD had a low negative correlation with levels of self-esteem Self-esteem mediated a relationship between ADHD and suicidal ideation
Bae et al. (2019)	55	Students recruited from a university (Korea) High caffeine *n* = 92 (*M*_age_ = 22.74, 41.3% female). Control *n* = 419 (*M*_age_ = 22.96, 57.0% female)	Cross-sectional—quantitative between-groups survey	K-AADHDS	Symptom severity of ADHD had a low negative correlation with levels of self-esteem
Chamberlain et al. (2017)	73	Gamblers recruited from the media advertisement (USA) ADHD symptomatic *n* = 86 (*M*_age_ = 22.3; 37.2% female). Control *n* = 337 (*M*_age_ = 22.3, 36.8% female)	Cross-sectional—quantitative between-subject design	ASRS 1.1	Participants with symptomatic ADHD reported lower levels of self-esteem compared to controls Reported no effects of gender on the difference in self-esteem between symptomatic ADHD and controls
Dan & Raz (2015)	62	Recruited from a pool of undergraduate students based on ASRS and OCPT (Israel) ADHD *n* = 25 (*M*_age_ = 23.84; 100% female). Control = 30 (*M*_age_ = 23.17; 100% female)	Cross-sectional—quantitative survey and test design between groups	Diagnosed with adult ADHD by either a psychiatrist or neurologist. + ASRS1.1 and OCPT	Participants with diagnosed ADHD reported lower levels of self-esteem compared to controls Self-esteem mediated a relationship between ADHD and social derogation and between ADHD and cognitive obstruction Participants with inattentive presentation and combined presentation reported no significant differences in self-esteem
Evren et al. (2021)	64	Recruited from an inpatient alcohol use disorder clinic (Turkey) Sample *n* = 151 (*M*_age_ = 45.73, gender not reported)	Cross-sectional—quantitative survey and interview design	ASRS 1.1	Symptom severity of ADHD had a moderate negative correlation with levels of self-esteemSelf-esteem correlated higher with symptoms of inattentiveness than with symptoms of hyperactivity/impulsivitySelf-esteem mediated a relationship between ADHD and social anxiety disorder
Jhambh et al. (2014)	62	Convenience sampling of students from a university (India) ADHD symptomatic *n* = 13 (61.5% female). Control *n* = 224 (66.5% female). Age range total 18 to 25 years (*M*_age_ total = 19.46)	Cross-sectional—quantitative between-subjects design	ASRS 1.1, WURS	Significantly more participants with symptomatic ADHD (53.8%) scored below cut-off for poor self-esteem compared to controls (19.2%)
Masuch et al. (2019)	66	Recruited from ADHD treatment centers (78,8%) and self-help groups (21.2%) (Germany) ADHD *n* = 104 (*M*_age_ = 41.8; 54.8% female). No controls.	Cross-sectional – quantitative survey and correlational design	ADHD-SR (German)	37.5% of the diagnosed ADHD group scored below cut-off for poor self-esteem
Michielsen et al. (2014)	70	Recruited from Longitudinal Aging Study Amsterdam (LASA) (Netherlands) ADHD *n* = 23 (*M*_age_ = 68.0, 52.4% female). Control *n* = 208 (*M*_age_ = 72.0, 60.1% female)	Cross-sectional—quantitative between-subjects design	DIVA 2.0	Participants with diagnosed ADHD reported lower levels of self-esteem when controlled for age Symptom severity of ADHD had a low negative correlation with levels of self-esteem Self-esteem mediated a relationship between ADHD and depressive symptoms
Newark et al. (2016)	63	ADHD group recruited from an outpatient clinic. Control group was recruited using convenience sampling (Switzerland)ADHD *n* = 43 (*M*_age_ = 34.3, 44.2% female). Control *n* = 43 (*M*_age_ = 34.6, 44.2% female)	Cross-sectional—quantitative between-subjects design	Met DSM-IV criteria for ADHD in adulthoodDiagnosed by an experienced clinical psychologist through a structural clinical interview	Participants with diagnosed ADHD reported lower levels of self-esteem compared to controls
Pawaskar et al. (2020)	77	Identified by VALIDATE DATA survey (USA) ADHD *n* = 444 (*M*_age_ = 42.5, 46.6 % female). ADHD Symptomatic *n* = 1055 (*M*_age_ = 43.5, 53.1% female)	Cross-sectional—quantitative between-subjects design	ASRS 1.1	Participants with diagnosed ADHD reported higher levels of self-esteem compared to participants with symptomatic ADHD
Turel and Bechara (2016)	69	Students recruited from a university (USA) ADHD symptomatic *n* = 110. Control *n* = 347. (*M*_age_ total sample = 23.4, 50.85% female)	Time-lagged—quantitative survey	ASRS 1.1 (part A)	Symptom severity of ADHD has a low negative correlation with levels of self-esteem Self-esteem mediated a relationship between ADHD and craving to use social network sites while driving

*Note. n* = group size; *M* = mean; ADHD symptomatic = self-reported symptom severity level indicative of ADHD diagnosis; OCPT = online continuous performance test.

All studies reporting scores on the 10-item version of RSES within an ADHD group are presented in Table 2. Scores in control groups were included when available. Since the RSES is most frequently reported on a scale from 0 to 30, studies reported RSES scores on other scales were converted to a standard of 0 to 30, to facilitate comparability, using Equation (1).

(1) $$n_{\mathrm{RSES}}=\frac{o_{\mathrm{RSES}}-L_{\min}}{L_{\max}-L_{\min}}*30$$

Where $n_{\mathrm{RSES}}$ = New standardized RSES score; $o_{\mathrm{RSES}}$ = Original reported mean RSES; $L_{\min}$ = Likert minimum on original scale; and $L_{\max}$ = Likert maximum on original scale.

**Table 2.** Mean Scores Rosenberg Self-Esteem Scale.

Author and year	Diagnosed ADHD	Symptomatic ADHD	Control	*p* Value
Diagnosed ADHD				
Dan and Raz (2015)	18.6^a^		24.6^a^	<.001
Masuch et al. (2019)	17.0			
Newark et al. (2016)	15.0		23.2	<.001
Pawaskar et al. (2020)	19.3	15.17		<.001
ADHD symptomatic				
Chamberlain et al. (2017)		20.2	22.4	<.005
Turel and Bechara (2016)		19.65^a^	21.15^a^	<.05

a Converted RSES mean to scale from 0 to 30.

Means that were reported on a scale that did not start at zero, were subtracted by the minimum level score on the Likert scale, to start at zero. The new maximum Likert level score was calculated by subtracting the original minimum Likert level from the original maximum level. Thereafter, the new mean was divided by the new maximum level score of the Likert scale. Lastly, to convert the numbers to a standardized mean, ranging from 0 to 30, the transformed mean was multiplied by 30.

Although this transformation could imply methodological concerns that are of serious risk to the validity of the transformed RSES means, it provides an indication of the levels of self-esteem across the samples. The RSES scores that were transformed, are highlighted by an asterisk in Table 2.

## Results

### Study Selection

The search procedure is described in Figure 1, showing that the initial literature search returned 387 records. After removing duplicates (190) and non-eligible articles (186), five articles were excluded after full-text screening (Babinski et al., 2011; Davtian et al., 2012; Gudjonsson et al., 2014; Kirino et al., 2015; Romo et al., 2015). A total of eleven studies were included in this review.

### Quality Assessment

The quality assessment scores ranged from 55% to 77%, with a mean of 67% (see Supplemental Table 1 for detailed descriptions). Four studies obtained a quality score of 70% or above (Arsandaux et al., 2021; Chamberlain et al., 2017; Michielsen et al., 2014; Pawaskar et al., 2020), while the quality scores for five studies ranged from 55% to 64% (Bae et al., 2019; Dan & Raz, 2015; Evren et al., 2021; Jhambh et al., 2014; Newark et al., 2016) due to insufficient identification, justification, and control of potentially confounding variables. Regarding methodology, most studies did not conduct power analysis (*n* = 3), adjusted for multiple comparisons (*n* = 3), and compared characteristics of excluded and included participants (*n* = 1). No study stated to have implemented measures to avoid recall bias.

### Study Characteristics

All studies were published between 2014 and 2021 and included participants from countries across Europe (*n* = 4), North America (*n* = 3), Middle East (*n* = 2), and Asia (*n* = 2). Most studies (nine) used cross-sectional designs and eight of those included either a healthy control group (*n* = 7) or a symptomatic undiagnosed control group (*n* = 1). Self-esteem in adults with ADHD was explicitly described as a main research focus in five of the included studies (Arsandaux et al., 2021; Evren et al., 2021; Dan & Raz, 2015; Newark et al., 2016; Pawaskar et al., 2020), while the remaining studies investigated ADHD and self-esteem as part of a broader research question related to psychological and psychosocial factors.

### Sample Characteristics

Table 1 provides an overview of the sample size, gender distribution, definition of ADHD, and age. A total of 6,085 participants were included, with sample sizes ranging from 55 to 2,331. The mean age of the participants ranged from 19.46 to 71 years. Overall, more women (*n* = 3,729) than men (n = 2,205) were included in the 10 studies reporting gender. ADHD medication usage among participants was only reported in one study (Masuch et al., 2019). No studies included information regarding nonbinary participants. Race/ethnicity was only reported in the study by Pawaskar et al. (2020) and education level and employment status were reported in nine of the studies. Five studies included participants with a clinical ADHD diagnosis, while six studies defined ADHD based on self-report. Only one study reported the distribution of ADHD presentation within their sample (Dan & Raz, 2015).

### Comorbidities With ADHD

Only 4 of the 11 included studies assessed and reported the prevalence of one or more comorbid disorders in their participants with ADHD (Chamberlain et al., 2017; Jhambh et al., 2014; Newark et al., 2016; Pawaskar et al., 2020): anxiety disorder (*n* = 3), eating disorders (*n* = 3), substance abuse disorder (*n* = 3), depression (*n* = 2), OCD (*n* = 1), insomnia (*n* = 1), panic disorder (*n* = 1), and post-traumatic stress disorder (*n* = 1). Additionally, four other studies (Arsandaux et al., 2021; Evren et al., 2021; Masuch et al., 2019; Michielsen et al., 2014) reported the mean symptom level of psychiatric disorders for their participants: depression (*n* = 3), anxiety (*n* = 2), and somatic symptom disorder (*n* = 1).

### Level of Self-Esteem Among Adults With ADHD

Seven studies reported mean RSES scores for the ADHD groups (diagnosed or symptomatic). The six studies that used the 10-item version are presented in Table 2, which also includes two studies with converted means (marked with an asterisk). The mean RSES scores in the diagnosed ADHD groups varied between 15.0 and 19.3 (mean = 17.48 ± 1.91), while the mean RSES scores in the symptomatic ADHD groups varied between 15.2 and 20.2 (mean = 18.34 ± 2.76). RSES scores ranged from 21.2 to 24.6 (mean = 22.84 ± 1.45) in the control groups.

Table 2 reflects the large heterogeneity in reported levels of self-esteem between the studies, in the ADHD and the control groups. The lowest mean level of self-esteem was found in the diagnosed ADHD group included in Newark et al.’s (2016) study. The ADHD group in that study consisted of treatment-seeking individuals who were diagnosed at site and characterized by a high mean age, as well as a high frequency of mood disorders and psychological distress. The symptomatic control group in Pawaskar et al.’s (2020) study obtained the second lowest mean score on RSES, while their group of adults formerly diagnosed with ADHD at a clinical ward obtained one of the highest mean scores among the included studies. The highest self-esteem was reported in two samples of young (mean age 22.3 and 23.4) nontreatment-seeking adults with symptomatic ADHD (Chamberlain et al., 2017; Turel & Bechara, 2016).

Two studies reported the proportion with a score below the threshold for low self-esteem (RSES ≤ 15) in their ADHD groups. The proportion was 53.8% in Jhambh et al.’s (2014) study in a symptomatic ADHD group, and 37.5% in Masuch et al.’s (2019) study including a diagnosed ADHD group.

### Diagnosed ADHD Groups Versus Controls

Three studies compared the self-esteem level in a group of adults diagnosed with ADHD and a group of healthy controls. In a study of the relationship between ADHD, self-esteem, and test anxiety, Dan and Raz (2015) showed that the self-esteem in 25 female undergraduate students with ADHD (*M* = 4.10 ± 0.95) was significantly lower than in a group of 30 age-matched female controls (*M* = 5.10 ± 0.76, *p* = .001). Michielsen et al. (2014) performed a study of the relationships between ADHD, self-esteem, and depression in a group of older adults with ADHD (*n* = 23, *M*_age_ = 68.0 ± 4.9) and controls (*n* = 208, *M*_age_ = 72.0 ± 7.9). The differences in self-esteem between the ADHD (*M* = 13.87 ± 3.22) and control group (*M* = 14.85 ± 2.19) were non-significant (*p* = .17). However, when age was included as a covariate, the association between ADHD diagnosis and self-esteem became statistically significant *r* = −.20, *p* *=* .*024*). Lower self-esteem in an ADHD than in a control group (*M* = 15.0 ± 6.5 vs. *M* = 23.2 ± 6.5, *p* < .01) was also found in Newark et al.’s (2016) study.

### Symptomatic ADHD Versus Controls

Three studies compared the self-esteem level of adults with symptomatic ADHD to healthy controls. Chamberlain et al. (2017) investigated self-esteem in a symptomatically defined ADHD group (*n* = 86, *M*_age_ = 22.3) and a healthy control group (*n* = 33.7 *M*_age_ = 22.3), recruited based on gambling behavior. The group with symptomatic ADHD displayed significantly lower self-esteem compared to healthy controls (*M* = 20.2 ± 6.0 vs. *M* = 22.4 ± 6.3, *p* = .005, *d* = 0.36). Jhambh et al. (2014) investigated the prevalence of ADHD and its relationship to low self-esteem (RSES ≤ 15) in students (age range = 18–25, *M*_age_ = 19.5) and found that a symptomatic ADHD group (*n* = 13) showed significantly lower self-esteem than students without ADHD (*n* = 224; *p* = .008). Turel and Bechara (2016) investigated whether self-esteem could partly mediate the relationship between ADHD symptoms and the use of social network sites while driving (*M*_age_ = 23.4). The students in the ADHD symptomatic group (*n* = 110) showed significantly lower self-esteem than students in a healthy control group (*n* = 347, *M* = 19.7 vs. *M* = 21.2, *p* < .05).

### Diagnosed Versus Symptomatic ADHD

Pawaskar et al. (2020) sought to explore the effects of diagnosis in adults with symptoms of ADHD. When comparing adults with symptomatic (*n* = 867, *M*_age_ = 43.5) and diagnosed ADHD (*n* = 436, *M*_age_ = 42.5), the latter group reported significantly higher self-esteem ratings (*M* = 19.3 ± 6.6 vs. *M* = 15.2 ± 6.3, *p* < .001).

### Association Between Symptoms of ADHD and Self-Esteem

Three studies showed negative correlations between the presence or severity of ADHD symptoms and self-esteem. Bae et al. (2019) found a weak negative correlation between the presence of ADHD symptoms and self-esteem (*r* = −.27, *p* < .01.) in students with high caffeine consumption (*n* = 92, *M*_age_ = 22.7), and controls (*n* = 419, *M*_age_ = 23.0). Michielsen et al. (2014) found a significant negative correlation between self-esteem in the presence of an ADHD diagnosis (*r* = −.20, *p* = .02) and between ADHD symptoms and self-esteem (*r* = −.25, *p* = .002) regardless of group status, while Turel and Bechara (2016) found a weak, but statistically significant negative correlation between ADHD symptoms and self-esteem in a student sample (*n* = 457, *r* = −.26, *p* < .01). In addition, Evren at al. (2021) reported a moderate correlation between ADHD symptoms and self-esteem (*r* = .421, *p* < .001) in a sample consisting of individuals with alcohol use disorder.

### Impact of Demographic and Clinical Characteristics

The few studies analyzing the impact of gender reported no differences between men and women (Chamberlain et al., 2017; Newark et al., 2016). The differences were also non-significant for the inattentive and combined ADHD presentations in the female sample included in Dan and Raz’s (2015) study, where 52% were classified with an inattentive, 44% with a combined, and 4% with a hyperactive-impulsive presentation. Notably, Evren et al. (2021) showed that self-esteem was associated with inattentive symptoms (*r* = .44, *p* < .001) and hyperactivity/impulsivity symptoms (*r* = .30, *p* < .001) in a sample of inpatients with alcohol use disorder. However, it was only the inattentive dimension that was predicted by self-esteem in a MANCOVA analysis. Newark et al. (2016) highlighted psychological distress as a potential contributor to group differences in self-esteem. When analyzing the effect of psychological distress on self-esteem, they found a higher level of psychological distress in the ADHD group than in a group of healthy controls, but the correlation between general psychological distress and level of self-esteem was statistically significant in both groups (*r* = −.44, *p* < .01, vs. *r* = −.50, *p* < .01).

### Self-Esteem as a Mediator or Moderator

Five studies investigated self-esteem as a mediator between ADHD symptoms and various life outcomes. With age as a covariate, Michielsen et al. (2014) found that mastery and self-esteem partly mediated the relationship between ADHD symptoms and depressive symptoms (*R*^2^ = .27) in a single mediation analysis. Arsandaux et al. (2021), who analyzed three different pathways from ADHD symptoms to suicidal ideation, found a mediating effect of self-esteem accounting for 45% of the association, compared to 25% through depression. A third pathway, through self-esteem and then depressive symptoms, accounted for an additional 19%. Their model adjusted for several covariates including age, gender, education level, conditions during college years, and substance consumption. Dan and Raz (2015), who analyzed the mediating effect of self-esteem on the relationship between ADHD and test anxiety using the Sobel test, found that the level of self-esteem functioned as a full mediator between ADHD and the social derogation facet of test anxiety (Sobel = −3.46, *p* = .0005) and as a partial mediator between ADHD and the cognitive obstruction facet of test anxiety (Sobel = −2.62, *p* = .008). The mediating role of self-esteem and harm avoidance on the relationship between symptoms of ADHD and symptoms of social anxiety was also studied by Evren et al. (2021). They found that self-esteem and harm avoidance together fully mediated the association between ADHD symptoms and symptoms of social anxiety [*F*(3,147) = 19.63, *p* < 0.001; Adjusted *R*^2^ = .27]. Finally, the study by Turel and Bechara (2016) should be mentioned. They investigated the potential mediating effects of stress and self-esteem on the relationship between ADHD symptoms and the use of social network sites (SNS) while driving. The relationship between ADHD and craving to use SNS was partially mediated by stress (*R*^2^ = .35) and self-esteem (*R*^2^ = .19), supporting that the level of self-esteem in ADHD may be a risk factor for use of SNS while driving.

## Discussion

The present systematic review aimed to identify and summarize the empirical literature analyzing self-esteem in adults with ADHD, with a focus on the potential impact of demographics (age, gender, race/ethnicity, education level, and employment status) and clinical characteristics (presentation of ADHD, symptom severity, and comorbid disorders). Methodological issues in this body of work were also identified as well as crucial gaps that exist in the literature. In sum, all studies in the current review support reduced self-esteem in adults with ADHD. The results also supported that self-esteem acts as a mediator in numerous negative life outcomes associated with adult ADHD. However, several methodological constraints were identified that limit firm conclusions.

In alignment with findings in the review by Cook et al. (2014), findings from the present study support a relationship between ADHD and reduced self-esteem in adults. This finding was well supported by the controlled studies, where four of five studies that compare an ADHD group to healthy controls found significantly lower self-esteem in the ADHD groups (Chamberlain et al., 2017; Dan & Raz, 2015; Newark et al., 2016; Turel & Bechara, 2016). Additionally, when controlling for age as a covariate, Michielsen et al. (2014) found that the participants with an ADHD diagnosis had significantly lower self-esteem than healthy controls. Overall, our findings strongly suggest that individuals with diagnosed ADHD, or who have symptoms indicative of ADHD, have lower self-esteem than in the general population. Furthermore, the severity of ADHD symptoms was negatively associated with self-esteem (Bae et al., 2019; Evren et al., 2021; Michielsen et al., 2014; Turel & Bechara, 2016), which further strengthening that adults with ADHD are at greater risk for low self-esteem.

Notably, low self-esteem was not restricted to adults with a formal ADHD diagnosis. Self-esteem was also impaired in adults with symptomatic ADHD (Chamberlain et al., 2017; Pawaskar et al., 2020; Turel & Bechara, 2016). This suggests that a diagnosed sample is not necessary to find group differences in self-esteem compared to healthy controls. Surprisingly, Pawaskar et al. (2020) found that adults diagnosed with ADHD were more likely to display higher self-esteem than symptomatic adults, potentially implying that living with the core symptoms of ADHD, not living with the diagnostic effect (e.g., stigma), is inflicting the most harm on self-esteem.

The review also supported an influencing role of demographic and clinical characteristics on self-esteem in adults with ADHD. For example, Michielsen et al. (2014) studied an older sample and observed no self-esteem differences between ADHD and a younger control group. After including age as a covariate, however, the ADHD group showed significantly lower self-esteem. This pattern is in accordance with studies showing that higher age is associated with reduced self-esteem (Orth et al, 2018; Orth & Robins, 2014).

Interestingly, gender differences appeared to be of minor importance regarding self-esteem in adults with ADHD (Chamberlain et al., 2017; Newark et al., 2016). By this, the literature review supported findings in previous reviews (Cook et al., 2014; Harpin et al., 2016) showing that males and females are more similar than different when it comes to self-esteem. The lack of observed gender differences is interesting, because men in the general population display slightly higher self-esteem than females (Orth et al., 2010), possibly explained by wording-effects where women tend to agree more easily with negatively worded self-statements (Magee & Upenieks, 2019). Based on two other studies, our findings suggest that all ADHD presentations are at risk of reduced self-esteem (Dan & Raz, 2015; Evren et al., 2021), but that the inattentive presentation may be at slightly higher risk (Evren et al., 2021). However, future research on demographic and clinical characteristics is needed given the limited number of studies.

It is also important to note that the relationship between self-esteem and ADHD is expected to be bidirectional: while low self-esteem can exacerbate difficulties related to ADHD symptoms (e.g., depression, social anxiety, and suicidal ideation), level of ADHD symptoms may also contribute to low self-esteem. This is supported by studies reporting negative correlations between severity level of ADHD symptoms and self-esteem (Bae et al., 2019; Evren et al., 2021; Michielsen et al., 2014; Turel & Bechara, 2016). This highlights the complexity of ADHD’s impact on self-esteem and vice versa, and the importance of addressing self-esteem in a therapeutic and clinical relationship.

Research unequivocally supports that ADHD is commonly associated with comorbid conditions like anxiety and depression (Katzman et al., 2017), each capable of independently influencing self-esteem (Sowislo & Orth, 2013). Conversely, self-esteem may influence the onset of these conditions in ADHD. However, given the correlational design of the reviewed studies, causal inferences or clarification of the nature of these relationships was not possible. Newark et al. (2016) found a moderate negative correlation between psychological distress and self-esteem in adults with ADHD, suggesting that psychological distress could moderate or mediate the negative correlation with self-esteem in adults with ADHD. Additionally, self-esteem has been explored as a mediator in the relationship between ADHD and associated mental health issues, such as depression (Michielsen et al., 2014), test anxiety (Dan & Raz, 2015), and social anxiety (Evren et al., 2021), with some evidence that self-esteem may play a more significant role than depression in the link between ADHD symptoms and suicidal ideation (Arsandaux et al., 2021).

The present systematic review has highlighted the reciprocal relationship between self-esteem and overall functioning in adults with ADHD, and thus the vital role of recognizing self-esteem in both mental health research and clinical practice. Furthermore, treatment programs may benefit from including self-esteem interventions, as outlined by U. de la Barrera et al. (2022). The importance of this issue is further highlighted by studies linking self-esteem to self-harm and suicidal ideation (Lippo et al., 2022), and by studies showing the impact of suicidal ideation on core aspects of the life of an adult with ADHD (Austgulen et al., 2023). Future studies should thus focus on designing studies that substantiate the significance of self-esteem on the overall functioning of adults with ADHD.

### Methodological Issues and Gaps in the Literature

In the present review, the results indicate a general lack of reporting and controlling of important demographic and clinical variables such as employment status, ethnicity, presentation and severity of ADHD, and comorbid disorders. Given that previous research supports that these factors are associated with self-esteem, future research would benefit from addressing these variables. Considering the widespread usage of medication in the ADHD population and the possible influence of medication on self-esteem (Biederman et al., 2004), it is a concern that only one study reported medication usage among ADHD participants. It is also noteworthy that zero studies reported on non-binary gender, as non-binary adults may suffer from reduced life-satisfaction (Kennis et al., 2022). The heterogeneity of the ADHD population, the varied methods of assessing ADHD, and the frequent use of student samples, pose challenges to generalizability. Furthermore, few studies adhere to rigorous methodological standards such as conducting power analysis, reporting effect sizes, correcting for multiple comparisons, or preventing recall bias. These methodological shortcomings leave the validity and generalizability of our findings open for questions.

Information about potential mediating or moderating factors with impact on the relationship between different presentations of ADHD and self-esteem is also still limited, and few studies have investigated why adults with ADHD exhibit lower self-esteem. It thus seems essential to develop and empirically evaluate theoretical frameworks (e.g., Young & Bramham, 2006) that can elucidate the relationship between ADHD and self-esteem. Such work will be critical both when assessing and personalizing intervention strategies. Taken together, there are still crucial gaps in the literature that restrict our understanding of the nature of the relationship between adult ADHD and self-esteem.

### Strengths and Limitations

The application of the PRISMA approach and the use of a quality assessment tool are strengths of the present study. Limiting our review to studies using RSES enhances comparability but also excludes other relevant studies. In that RSES assesses general self-esteem rather than domain-specific self-esteem, the included studies may also have missed the complexity of self-esteem dynamics in individuals with ADHD. The exclusion of treatment studies may also have led to the omission of potentially relevant research. Further, the psychometric properties of RSES for adults with ADHD are underexplored, raising concerns about possible wording effects. Converting RSES scores for consistency may lead to information loss. Lastly, the restriction to English-language articles introduces potential publication and cultural biases.

### Future Directions

In light of the findings of the present study, future research should continue to explore the relationship between self-esteem and ADHD. At present, most studies are correlational and cross-sectional, and longitudinal designs are crucial for understanding casual relationships and informing intervention strategies. Given the noted methodological issues, future studies should attempt to account for both demographic and clinical variables. It is essential that research include a broader range of demographic factors (e.g., gender, presentation, and comorbidity), and explore self-esteem among individuals formally diagnosed with ADHD and those with ADHD symptomatology. Furthermore, the applicability and validity of the RSES for adults with ADHD are unexplored and warrant investigation.

## Conclusion

The results of this study underscore a strong association between low self-esteem and ADHD, aligning with results from prior reviews. The synthesis of the literature not only supported the presence of low self-esteem in adults with an ADHD diagnosis, but also in those with symptom severity indicative of ADHD. Self-esteem was also found to be clinically important by being a mediator in the association between adult ADHD and several adverse outcomes, including depression, social anxiety, and suicidal ideation. Notably, several methodological issues characterize this body of research, which strongly encourage future research to identify mediating and moderating factors on self-esteem in adults with ADHD. The present systematic review also highlights the importance of the intersection between self-esteem, psychosocial functioning, and ADHD symptoms that may keep adults with ADHD in a vicious behavioral and emotional cycle. These findings support that self-esteem may be an important target for intervention for quality of life and to help diminish the severity of negative ADHD-related outcomes.

## Supplemental Material

sj-docx-1-jad-10.1177_10870547241237245 – Supplemental material for Self-Esteem in Adults With ADHD Using the Rosenberg Self-Esteem Scale: A Systematic Review Supplemental material, sj-docx-1-jad-10.1177_10870547241237245 for Self-Esteem in Adults With ADHD Using the Rosenberg Self-Esteem Scale: A Systematic Review by Aksel Bjørø Pedersen, Bernhard Vestby Edvardsen, Salvatore Matias Messina, Maria Rudjord Volden, Lisa L. Weyandt and Astri J. Lundervold in Journal of Attention Disorders

## References

[bibr1-10870547241237245] American Psychiatric Association. (2013). Diagnostic and statistical manual of mental disorders: DSM-5™ (5th ed.). American Psychiatric Publishing, Inc. 10.1176/appi.books.9780890425596

[bibr2-10870547241237245] Arsandaux J. Orri M. Tournier M. Gbessemehlan A. Coté S. Salamon R. Tzourio C. Galéra C. (2021). Pathways from ADHD symptoms to suicidal ideation during college years: A longitudinal study on the i-Share cohort [Journal Article; Research Support, Non-U.S. Gov’t]. Journal of Attention Disorders, 25(11), 1534–1543. 10.1177/1087054720915246 32338119

[bibr3-10870547241237245] Austgulen A. Skram N. K. G. Haavik J. Lundervold A. J. (2023). Risk factors of suicidal spectrum behaviors in adults and adolescents with attention-deficit / hyperactivity disorder: A systematic review. BMC Psychiatry, 23(1), 612. 10.1186/s12888-023-05099-8 37605105 PMC10441735

[bibr4-10870547241237245] Babinski D. E. Pelham W. E. Jr. Molina B. S. Waschbusch D. A. Gnagy E. M. Yu J. Sibley M. H. Biswas A. (2011). Women with childhood ADHD: Comparisons by diagnostic group and gender. Journal of Psychopathology and Behavioral Assessment, 33(4), 420–429. 10.1007/s10862-011-9247-4 22228922 PMC3251258

[bibr5-10870547241237245] Bae E. J. Kim E. B. Choi B. R. Won S. H. Kim J. H. Kim S. M. Yoo H. J. Bae S. M. Lim M. H. (2019). The relationships between addiction to highly caffeinated drinks, burnout, and Attention-Deficit/Hyperactivity Disorder. Journal of the Korean Academy of Child and Adolescent Psychiatry, 30(4), 153–160. 10.5765/jkacap.190015 32595336 PMC7298905

[bibr6-10870547241237245] Bemrose H. V. Akande I. O. Cullen A. E. (2021). Self-esteem in individuals at ultra-high risk for psychosis: A systematic review and meta-analysis. Early Intervention in Psychiatry, 15(4), 775–786. 10.1111/eip.13034 32860493

[bibr7-10870547241237245] Betancourt J. L. Alderson R. M. Roberts D. K. Bullard C. C. (2024). Self-esteem in children and adolescents with and without attention-deficit/hyperactivity disorder: A meta-analytic review. Clinical Psychology Review, 108, 102394. 10.1016/j.cpr.2024.102394 38286088

[bibr8-10870547241237245] Biederman J. Spencer T. Wilens T. (2004). Evidence-based pharmacotherapy for attention-deficit hyperactivity disorder. The International Journal of Neuropsychopharmacology, 7(1), 77–97. 10.1017/s1461145703003973 14733627

[bibr9-10870547241237245] Byth S. Frijters P. Beatton T. (2022). The relationship between obesity and self-esteem: longitudinal evidence from Australian adults. Oxford Open Economics, 1.

[bibr10-10870547241237245] Çelebi F. Ünal D. (2021). Self esteem and clinical features in a clinical sample of children with ADHD and social anxiety disorder. Nordic Journal of Psychiatry, 75(4), 286–291. 10.1080/08039488.2020.1850857 33475025

[bibr11-10870547241237245] Chamberlain S. R. Ioannidis K. Leppink E. W. Niaz F. Redden S. A. Grant J. E. (2017). ADHD symptoms in non-treatment seeking young adults: Relationship with other forms of impulsivity. CNS Spectrums, 22(1), 22–30. 10.1017/s1092852915000875 27680974 PMC5330410

[bibr12-10870547241237245] Chung W. Jiang S.-F. Paksarian D. Nikolaidis A. Castellanos F. X. Merikangas K. R. Milham M. P. (2019). Trends in the prevalence and incidence of Attention-Deficit/Hyperactivity Disorder among adults and children of different racial and ethnic groups. JAMA Network Open, 2(11), e1914344. 10.1001/jamanetworkopen.2019.14344 PMC6826640 31675080

[bibr13-10870547241237245] Colmsee I.-S. O. Hank P. Bošnjak M. (2021). Low self-esteem as a risk factor for eating disorders: A meta-analysis. Zeitschrift für Psychologie, 229(1), 48–69. 10.1027/2151-2604/a000433

[bibr14-10870547241237245] Cook J. Knight E. Hume I. Qureshi A. (2014). The self-esteem of adults diagnosed with attention-deficit/hyperactivity disorder (ADHD): A systematic review of the literature. Attention and Deficit Hyperactivity Disorders, 6(4), 249–268. 10.1007/s12402-014-0133-2 24668198

[bibr15-10870547241237245] Dan O. Raz S. (2015). The relationships among ADHD, self-esteem, and test anxiety in young adults. Journal of Attention Disorders, 19(3), 231–239. 10.1177/1087054712454571 22930792

[bibr16-10870547241237245] Davtian M. Reid R. C. Fong T. W. (2012). Investigating facets of personality in adult pathological gamblers with ADHD. Neuropsychiatry (London), 2(2), 163–174. 10.2217/npy.12.11 22815658 PMC3397924

[bibr17-10870547241237245] De la Barrera U. Montoya-Castilla I. Pérez-Albéniz A. Lucas-Molina B. Fonseca-Pedrero E. (2022). Mental health difficulties related to suicidal behavior in adolescents: The moderating role of self-esteem. Archives of Suicide Research, 26(2), 716–730. 10.1080/13811118.2020.1823918 33027593

[bibr18-10870547241237245] Donnellan M. B. Trzesniewski K. H. Robins R. W. (2015). Chapter 6: Measures of self-esteem. In Boyle G. J. Saklofske D. H. Matthews G. (Eds.), Measures of personality and social psychological constructs (pp. 131–157). Academic Press. 10.1016/B978-0-12-386915-9.00006-1

[bibr19-10870547241237245] Evren C. Cicekci E. Umut G. Evren B. Durmus Cicek K. (2021). The mediating effects of self-esteem and harm avoidance on the association between social anxiety symptoms and Adult Attention Deficit Hyperactivity Disorder Symptom severity in turkish inpatients with alcohol use disorder. Iranian Journal of Psychiatry, 16(3), 281–289. 10.18502/ijps.v16i3.6253 34616461 PMC8452829

[bibr20-10870547241237245] Faraone S. V. Biederman J. Mick E. (2006). The age-dependent decline of attention deficit hyperactivity disorder: A meta-analysis of follow-up studies. Psychological Medicine, 36(2), 159–165. 10.1017/S003329170500471X 16420712

[bibr21-10870547241237245] Franke B. Michelini G. Asherson P. Banaschewski T. Bilbow A. Buitelaar J. K. Cormand B. Faraone S. V. Ginsberg Y. Haavik J. Kuntsi J. Larsson H. Lesch K. P. Ramos-Quiroga J. A. Réthelyi J. M. Ribases M. Reif A. (2018). Live fast, die young? A review on the developmental trajectories of ADHD across the lifespan. European Neuropsychopharmacology, 28(10), 1059–1088. 10.1016/j.euroneuro.2018.08.001 30195575 PMC6379245

[bibr22-10870547241237245] Gjervan B. Hjemdal O. Nordahl H. M. (2016). Functional impairment mediates the relationship between adult ADHD inattentiveness and occupational outcome. Journal of Attention Disorders, 20(6), 510–518. 10.1177/1087054712474689 23407280

[bibr23-10870547241237245] Groves N. B. Wells E. L. Soto E. F. Marsh C. L. Jaisle E. M. Harvey T. K. Kofler M. J. (2022). Executive functioning and emotion regulation in children with and without ADHD. Research on Child and Adolescent Psychopathology, 50(6), 721–735. 10.1007/s10802-021-00883-0 34762251 PMC9091051

[bibr24-10870547241237245] Gudjonsson G. H. Sigurdsson J. F. Sigfusdottir I. D. Young S. (2014). A national epidemiological study of offending and its relationship with ADHD symptoms and associated risk factors. Journal of Attention Disorders, 18(1), 3–13. 10.1177/1087054712437584 22522573

[bibr25-10870547241237245] Halleland H. B. Haavik J. Lundervold A. J. (2012). Set-shifting in adults with ADHD. Journal of the International Neuropsychological Society, 18(4), 728–737. 10.1017/S1355617712000355 22613368

[bibr26-10870547241237245] Halleröd S. L. H. Anckarsäter H. Råstam M. Hansson Scherman M. (2015). Experienced consequences of being diagnosed with ADHD as an adult: A qualitative study. BMC Psychiatry, 15, 31. 10.1186/s12888-015-0410-4 PMC4376140 25884685

[bibr27-10870547241237245] Harpin V. Mazzone L. Raynaud J. P. Kahle J. Hodgkins P. (2016). Long-term outcomes of ADHD: A systematic review of self-esteem and social function. Journal of Attention Disorders, 20(4), 295–305. 10.1177/1087054713486516 23698916

[bibr28-10870547241237245] Henning C. Summerfeldt L. J. Parker J. D. A. (2022). ADHD and academic success in university students: The important role of impaired attention. Journal of Attention Disorders, 26(6), 893–901. 10.1177/10870547211036758 34384265 PMC8859654

[bibr29-10870547241237245] Hinshaw S. P. Nguyen P. T. O’Grady S. M. Rosenthal E. A. (2022). Annual research review: Attention-deficit/hyperactivity disorder in girls and women: Underrepresentation, longitudinal processes, and key directions. Journal of Child Psychology and Psychiatry, 63(4), 484–496. 10.1111/jcpp.13480 34231220

[bibr30-10870547241237245] Instanes J. T. Klungsøyr K. Halmøy A. Fasmer O. B. Haavik J. (2018). Adult ADHD and comorbid somatic disease: A systematic literature review. Journal of Attention Disorders, 22(3), 203–228. 10.1177/1087054716669589 27664125 PMC5987989

[bibr31-10870547241237245] Isomaa R. Väänänen J. M. Fröjd S. Kaltiala-Heino R. Marttunen M. (2013). How low is low? Low self-esteem as an indicator of internalizing psychopathology in adolescence. Health Education & Behavior, 40(4), 392–399. 10.1177/1090198112445481 22872582

[bibr32-10870547241237245] Jhambh I. Arun P. Garg J. (2014). Cross-sectional study of self-reported ADHD symptoms and psychological comorbidity among college students in Chandigarh, India. Industrial Psychiatry Journal, 23(2), 111–116. 10.4103/0972-6748.151679 25788800 PMC4361973

[bibr33-10870547241237245] Jordan C. H. (2020). Rosenberg Self-Esteem Scale. In Zeigler-Hill V. Shackelford T. K. (Eds.), Encyclopedia of personality and individual differences (pp. 4518–4520). Springer International Publishing. 10.1007/978-3-319-24612-3_1155

[bibr34-10870547241237245] Kashdan T. B. (2004). The assessment of subjective well-being (issues raised by the Oxford Happiness Questionnaire). Personality and Individual Differences, 36(5), 1225–1232. 10.1016/S0191-8869(03)00213-7

[bibr35-10870547241237245] Katzman M. A. Bilkey T. S. Chokka P. R. Fallu A. Klassen L. J. (2017). Adult ADHD and comorbid disorders: Clinical implications of a dimensional approach. BMC Psychiatry, 17, 302. 10.1186/s12888-017-1463-3 28830387 PMC5567978

[bibr36-10870547241237245] Kazda L. McGeechan K. Bell K. Thomas R. Barratt A. (2022). Association of Attention-Deficit/Hyperactivity Disorder diagnosis with adolescent quality of life. JAMA Network Open, 5(10), e2236364. 10.1001/jamanetworkopen.2022.36364 PMC9561944 36227598

[bibr37-10870547241237245] Kennis M. Duecker F. T’Sjoen G. Sack A. T. Dewitte M. (2022). Mental and sexual well-being in non-binary and genderqueer individuals. International Journal of Transgender Health, 23(4), 442–457. 10.1080/26895269.2021.1995801 36324878 PMC9621256

[bibr38-10870547241237245] Kirino E. Imagawa H. Goto T. Montgomery W. (2015). Sociodemographics, comorbidities, healthcare utilization and work productivity in Japanese patients with adult ADHD. PLoS One, 10(7), e0132233. 10.1371/journal.pone.0132233 PMC4493035 26147097

[bibr39-10870547241237245] Kita Y. Inoue Y. (2017). The direct/indirect association of ADHD/ODD symptoms with self-esteem, self-perception, and depression in early adolescents. Frontiers in Psychiatry, 8, 137. 10.3389/fpsyt.2017.00137 28824468 PMC5534463

[bibr40-10870547241237245] Kooij J. J. Michielsen M. Kruithof H. Bijlenga D. (2016). ADHD in old age: A review of the literature and proposal for assessment and treatment. Expert Review of Neurotherapeutics, 16(12), 1371–1381. 10.1080/14737175.2016.1204914 27334252

[bibr41-10870547241237245] Kooij J. J. S. Bijlenga D. Salerno L. Jaeschke R. Bitter I. Balázs J. Thome J. Dom G. Kasper S. Nunes Filipe C. Stes S. Mohr P. Leppämäki S. Casas M. Bobes J. McCarthy J. M. Richarte V. Kjems Philipsen A. Pehlivanidis A. . . . Asherson P. (2019). Updated European Consensus Statement on diagnosis and treatment of adult ADHD. European Psychiatry, 56, 14–34. 10.1016/j.eurpsy.2018.11.001 30453134

[bibr42-10870547241237245] Kooij S. J. Bejerot S. Blackwell A. Caci H. Casas-Brugué M. Carpentier P. J. Edvinsson D. Fayyad J. Foeken K. Fitzgerald M. Gaillac V. Ginsberg Y. Henry C. Krause J. Lensing M. B. Manor I. Niederhofer H. Nunes-Filipe C. Ohlmeier M. D. . . . Asherson P. (2010). European consensus statement on diagnosis and treatment of adult ADHD: The European Network Adult ADHD. BMC Psychiatry, 10, 67. 10.1186/1471-244x-10-67 20815868 PMC2942810

[bibr43-10870547241237245] Kuster F. Orth U. Meier L. L. (2013). High self-esteem prospectively predicts better work conditions and outcomes. Social Psychological and Personality Science, 4(6), 668–675. 10.1177/1948550613479806

[bibr44-10870547241237245] Lippo F. Madeddu F. Fornaro M. Calati R. (2022). The association between self-esteem and suicidal risk: A meta-analysis. European Psychiatry, 65(S1), S835–S835. 10.1192/j.eurpsy.2022.2162

[bibr45-10870547241237245] Lundervold A. J. Halleland H. B. Brevik E. J. Haavik J. Sørensen L. (2019). Verbal memory function in intellectually well-functioning adults with ADHD: Relations to working memory and response inhibition. Journal of Attention Disorders, 23(10), 1188–1198. 10.1177/1087054715580842 25903587

[bibr46-10870547241237245] Lynum L. I. Wilberg T. Karterud S. (2008). Self-esteem in patients with borderline and avoidant personality disorders. Scandinavian Journal of Psychology, 49(5), 469–477. 10.1111/j.1467-9450.2008.00655.x 18564322

[bibr47-10870547241237245] Magee W. Upenieks L. (2019). Gender differences in self-esteem, unvarnished self-evaluation, future orientation, self-enhancement and self-derogation in a U.S. national sample. Personality and Individual Differences, 149, 66–77. 10.1016/j.paid.2019.05.016

[bibr48-10870547241237245] Masuch T. V. Bea M. Alm B. Deibler P. Sobanski E. (2019). Internalized stigma, anticipated discrimination and perceived public stigma in adults with ADHD. Attention Deficit and Hyperactivity Disorders, 11(2), 211–220. 10.1007/s12402-018-0274-9 30341693

[bibr49-10870547241237245] Michielsen M. Comijs H. C. Semeijn E. J. Beekman A. T. F. Deeg D. J. H. Kooij J. J. S. (2014). Attention Deficit Hyperactivity Disorder and personality characteristics in older adults in the general dutch population. The American Journal of Geriatric Psychiatry, 22(12), 1623–1632. 10.1016/j.jagp.2014.02.005 24656507

[bibr50-10870547241237245] Munro B. A. Weyandt L. L. Hall L. E. Oster D. R. Gudmundsdottir B. G. Kuhar B. G. (2018). Physiological substrates of executive functioning: A systematic review of the literature. ADHD Attention Deficit and Hyperactivity Disorders, 10(1), 1–20. 10.1007/s12402-017-0226-9 28332146

[bibr51-10870547241237245] Newark P. Elsässer M. Stieglitz R.-D. (2016). Self-esteem, self-efficacy, and resources in adults with ADHD. Journal of Attention Disorders, 20, 279–290. 10.1177/1087054712459561 23074301

[bibr52-10870547241237245] Nutt D. J. Fone K. Asherson P. Bramble D. Hill P. Matthews K. Morris K. A. Santosh P. Sonuga-Barke E. Taylor E. Weiss M. Young S. , & British Association for, P. (2007). Evidence-based guidelines for management of attention-deficit/hyperactivity disorder in adolescents in transition to adult services and in adults: Recommendations from the British Association for Psychopharmacology. Journal of Psychopharmacology, 21(1), 10–41. 10.1177/0269881106073219 17092962

[bibr53-10870547241237245] Orth U. Erol R. Y. Luciano E. C. (2018). Development of self-esteem from age 4 to 94 years: A meta-analysis of longitudinal studies. Psychological Bulletin, 144(10), 1045–1080. 10.1037/bul0000161 30010349

[bibr54-10870547241237245] Orth U. Robins R. W. (2014). The development of self-esteem. Current Directions in Psychological Science, 23(5), 381–387. 10.1177/0963721414547414

[bibr55-10870547241237245] Orth U. Robins R. W. Widaman K. F. (2012). Life-span development of self-esteem and its effects on important life outcomes. Journal of Personality and Social Psychology, 102(6), 1271–1288. 10.1037/a0025558 21942279

[bibr56-10870547241237245] Orth U. Trzesniewski K. H. Robins R. W. (2010). Self-esteem development from young adulthood to old age: A cohort-sequential longitudinal study. Journal of Personality and Social Psychology, 98(4), 645–658. 10.1037/a0018769 20307135

[bibr57-10870547241237245] Page M. J. McKenzie J. E. Bossuyt P. M. Boutron I. Hoffmann T. C. Mulrow C. D. Shamseer L. Tetzlaff J. M. Akl E. A. Brennan S. E. Chou R. Glanville J. Grimshaw J. M. Hróbjartsson A. Lalu M. M. Li T. Loder E. W. Mayo-Wilson E. McDonald S. . . . Moher D. (2021). The PRISMA 2020 statement: An updated guideline for reporting systematic reviews. BMJ, 372, n71. 10.1136/bmj.n71 PMC8005924 33782057

[bibr58-10870547241237245] Paulson J. F. Buermeyer C. Nelson-Gray R. O. (2005). Social rejection and ADHD in young adults: An analogue experiment. Journal of Attention Disorders, 8(3), 127–135. 10.1177/1087054705277203 16009661

[bibr59-10870547241237245] Pawaskar M. Fridman M. Grebla R. Madhoo M. (2020). Comparison of quality of life, productivity, functioning and self-esteem in adults diagnosed with ADHD and with symptomatic ADHD. Journal of Attention Disorders, 24(1), 136–144. 10.1177/1087054719841129 31043096 PMC6935829

[bibr60-10870547241237245] Pinho T. D. Manz P. H. DuPaul G. J. Anastopoulos A. D. Weyandt L. L. (2019). Predictors and moderators of quality of life among college students with ADHD. Journal of Attention Disorders, 23(14), 1736–1745. 10.1177/1087054717734645 28992747 PMC6209539

[bibr61-10870547241237245] Polanczyk G. de Lima M. S. Horta B. L. Biederman J. Rohde L. A. (2007). The worldwide prevalence of ADHD: A systematic review and metaregression analysis. Am J Psychiatry, 164(6), 942-948. 10.1176/ajp.2007.164.6.942 17541055

[bibr62-10870547241237245] Richman G. Hope T. Mihalas S. (2010). Assessment and treatment of self-esteem in adolescents with ADHD. In Guindon M. H. (Ed.), Self-esteem across the lifespan: Issues and interventions (pp. 111–123). Routledge/Taylor & Francis Group.

[bibr63-10870547241237245] Robins R. W. Hendin H. M. Trzesniewski K. H. (2001). Measuring global self-esteem: Construct validation of a single-item measure and the Rosenberg Self-Esteem Scale. Personality and Social Psychology Bulletin, 27(2), 151–161. 10.1177/0146167201272002

[bibr64-10870547241237245] Romo L. Rémond J. J. Coeffec A. Kotbagi G. Plantey S. Boz F. Kern L. (2015). Gambling and Attention Deficit Hyperactivity Disorders (ADHD) in a population of French students. Journal of Gambling Studies, 31(4), 1261–1272. 10.1007/s10899-014-9515-9 25466366

[bibr65-10870547241237245] Rosenberg M. (1965). Society and the adolescent self-image. Princeton University Press.

[bibr66-10870547241237245] Rosenberg M. (1979). Conceiving the self. Basic Books.

[bibr67-10870547241237245] Rosenberg M. Simmons R. G. (1972). Black and White self-esteem: The urban school child. American Sociological Associaton.

[bibr68-10870547241237245] Šare S. Ljubičić M. Gusar I. Čanović S. Konjevoda S. (2021). Self-esteem, anxiety, and depression in older people in nursing homes. Healthcare (Basel, Switzerland), 9(8), 1035. 10.3390/healthcare9081035 34442172 PMC8392518

[bibr69-10870547241237245] Schiweck C. Arteaga-Henriquez G. Aichholzer M. Edwin Thanarajah S. Vargas-Cáceres S. Matura S. Grimm O. Haavik J. Kittel-Schneider S. Ramos-Quiroga J. A. Faraone S. V. Reif A. (2021). Comorbidity of ADHD and adult bipolar disorder: A systematic review and meta-analysis. Neuroscience & Biobehavioral Reviews, 124, 100–123. 10.1016/j.neubiorev.2021.01.017 33515607

[bibr70-10870547241237245] Schmitt D. P. Allik J. (2005). Simultaneous administration of the Rosenberg Self-Esteem Scale in 53 nations: Exploring the universal and culture-specific features of global self-esteem. Journal of Personality and Social Psychology, 89(4), 623–642. 10.1037/0022-3514.89.4.623 16287423

[bibr71-10870547241237245] Simon V. Czobor P. Bálint S. Mészáros Á. Bitter I. (2009). Prevalence and correlates of adult attention-deficit hyperactivity disorder: Meta-analysis. The British Journal of Psychiatry, 194(3), 204–211. 10.1192/bjp.bp.107.048827 19252145

[bibr72-10870547241237245] Song P. Zha M. Yang Q. Zhang Y. Li X. Rudan I. (2021). The prevalence of adult attention-deficit hyperactivity disorder: A global systematic review and meta-analysis. Journal of Global Health, 11, 04009. 10.7189/jogh.11.04009 33692893 PMC7916320

[bibr73-10870547241237245] Sowislo J. F. Orth U. (2013). Does low self-esteem predict depression and anxiety? A meta-analysis of longitudinal studies. Psychological Bulletin, 139(1), 213–240. 10.1037/a0028931 22730921

[bibr74-10870547241237245] Thomas R. Sanders S. Doust J. Beller E. Glasziou P. (2015). Prevalence of Attention-Deficit/Hyperactivity Disorder: A systematic review and meta-analysis. Pediatrics, 135(4), e994–e1001. 10.1542/peds.2014-3482 25733754

[bibr75-10870547241237245] Torgersen T. Gjervan B. Rasmussen K. (2006). ADHD in adults: A study of clinical characteristics, impairment and comorbidity. Nordic Journal of Psychiatry, 60(1), 38–43. 10.1080/08039480500520665 16500798

[bibr76-10870547241237245] Trzesniewski K. H. Donnellan M. B. Moffitt T. E. Robins R. W. Poulton R. Caspi A. (2006). Low self-esteem during adolescence predicts poor health, criminal behavior, and limited economic prospects during adulthood. Developmental Psychology, 42(2), 381–390. 10.1037/0012-1649.42.2.381 16569175

[bibr77-10870547241237245] Turel O. Bechara A. (2016). Social networking site use while driving: ADHD and the mediating roles of stress, self-esteem and craving. Frontiers in Psychology, 7, 455. 10.3389/fpsyg.2016.00455 27065923 PMC4812103

[bibr78-10870547241237245] Twenge J. M. Campbell W. K. (2002). Self-esteem and socioeconomic status: A meta-analytic review. Personality and Social Psychology Review, 6(1), 59–71. 10.1207/S15327957PSPR0601_3

[bibr79-10870547241237245] Twenge J. M. Crocker J. (2002). Race and self-esteem: Meta-analyses comparing Whites, Blacks, Hispanics, Asians, and American Indians and comment on Gray-Little and Hafdahl (2000). Psychological Bulletin, 128(3), 371–408. 10.1037/0033-2909.128.3.371 12002695

[bibr80-10870547241237245] von Soest T. Wagner J. Hansen T. Gerstorf D . (2018). Self-esteem across the second half of life: The role of socioeconomic status, physical health, social relationships, and personality factors. Journal of Personality and Social Psychology, 114(6), 945–958. 10.1037/pspp0000123 28150978

[bibr81-10870547241237245] Weyandt L. L. Oster D. R. Gudmundsdottir B. G. DuPaul G. J. Anastopoulos A. D. (2017). Neuropsychological functioning in college students with and without ADHD. Neuropsychology, 31(2), 160–172. 10.1037/neu0000326 27831696 PMC5280458

[bibr82-10870547241237245] Willcutt E. G. (2012). The prevalence of DSM-IV Attention-deficit/Hyperactivity Disorder: A meta-analytic review. Neurotherapeutics, 9(3), 490–499. 10.1007/s13311-012-0135-8 22976615 PMC3441936

[bibr83-10870547241237245] Young S. Bramham J. (2006). ADHD in adults: A psychological guide to practice (1st ed.). Wiley Hoboken. http://public.eblib.com/choice/publicfullrecord.aspx?p=281585

