# Supplementary material for: Self-Esteem in Adults With ADHD Using the Rosenberg Self-Esteem Scale: A Systematic Review
Source: J Atten Disord. 2024 Mar 16;28(7):1124–38. doi: 10.1177/10870547241237245 (PMC11016209; doi:10.1177/10870547241237245)
Supplement: sj-docx-1-jad-10.1177_10870547241237245 – Supplemental material for Self-Esteem in Adults With ADHD Using the Rosenberg Self-Esteem Scale: A Systematic Review [file sj-docx-1-jad-10.1177_10870547241237245.docx]

| **Supplementary Table 1.** Quality assessment results | | | | |
| --- | --- | --- | --- | --- |
|  | Arsandaux et al. (2021) | Bae et al. (2019) | Chamberlain et al. (2017) | Dan & Raz (2015) |
| 1. Background described | Yes | Yes | Yes | Yes |
| 2. Study design reported | Yes | Yes | Yes | Yes |
| 3. Main outcomes described | Yes | Yes | Yes | Yes |
| 4. Aims and objectives reported | Yes | Yes | Yes | Yes |
| 5. Sources and methods of recruitment described | Yes | Yes | Yes | Partially |
| 6. Participant characteristics described | Yes | Yes | Yes | Partially |
| 7. Method of diagnosis based on standardized definition | Yes | Yes | Yes | Yes |
| 8. Sample representative of target population | No | No | Partially | Partially |
| 9. Inclusion/exclusion criteria | Yes | No | Yes | Yes |
| 10. Excluded/lost to follow-up information reported | Yes | Partially | No | Yes |
| 11. Power calculation used | No | No | Yes | No |
| 12. Comparison between excluded and included participants | Yes | No | No | No |
| 13. Adequate controls | N/A | N/A | Yes | Yes |
| 14. Age/stage specified in relation to diagnosis | Can't tell | Can't tell | Can't tell | Partially |
| 15. Data extraction specified | Yes | Yes | Yes | Yes |
| 16. Variables of interest defined | Yes | Yes | Yes | Yes |
| 17. Reliable measurement of self-esteem | Yes | Yes | Yes | Yes |
| 18. Assessment of ADHD subtype | No | Yes | No | Yes |
| 19. Error due to recall bias avoided | No | No | No | Can't tell |
| 20. Researchers blind to ADHD diagnosis/treatment status | Can't tell | Can't tell | Can't tell | Can't tell |
| 21. Potential confounders defined | Yes | Partially | Yes | No |
| 22. Inclusion of potential confounders justified | Yes | Partially | Yes | No |
| 23. Methods taken to control confounding | Yes | No | Yes | No |
| 24. Large enough sample for statistics | Can't tell | Can´t tell | Yes | Can't tell |
| 25. Multiple comparison corrections | No | No | No | No |
| 26. Statistical methods chosen are suitable | Yes | Yes | Yes | Yes |
| 27. Relevant factors included in analysis | Yes | Partially | Partially | Yes |
| 28. Generalizability of the study results discussed | Yes | Yes | Yes | Partially |
| 29. Declaration of conflict of interest? | Yes | Yes | Yes | Yes |
| 30. Sources of funding identified | Yes | No | Yes | Yes |
| 31. Percentage | 72% | 55% | 73% | 62% |
|  |  |  |  |  |
| **Supplementary Table 1.**  **(continued)** | | | | |
|  | Evren et al. (2021) | Jhambh et al. (2014) | Masuch et al. (2018) | Michielsen et al. (2014) |
| 1. Background described | Yes | Yes | Yes | Yes |
| 2. Study design reported | Yes | Yes | Yes | Yes |
| 3. Main outcomes described | Yes | Yes | Yes | Yes |
| 4. Aims and objectives reported | Yes | Yes | Yes | Yes |
| 5. Sources and methods of recruitment described | Yes | Yes | Yes | Yes |
| 6. Participant characteristics described | Partially | Yes | Yes | Partially |
| 7. Method of diagnosis based on standardized definition | Yes | Yes | Yes | Yes |
| 8. Sample representative of target population | No | Partially | Yes | Partially |
| 9. Inclusion/exclusion criteria | Yes | Yes | Yes | Yes |
| 10. Excluded/lost to follow-up information reported | Yes | Yes | Yes | Yes |
| 11. Power calculation used | Yes | No | No | Yes |
| 12. Comparison between excluded and included participants | No | No | No | No |
| 13. Adequate controls | N/A | Yes | N/A | Yes |
| 14. Age/stage specified in relation to diagnosis | Can't tell | Can't tell | No | Partially |
| 15. Data extraction specified | Yes | Yes | Yes | Yes |
| 16. Variables of interest defined | Yes | Yes | Yes | Yes |
| 17. Reliable measurement of self-esteem | Yes | Yes | Yes | Yes |
| 18. Assessment of ADHD subtype | Yes | No | No | No |
| 19. Error due to recall bias avoided | No | No | Can't tell | Can't tell |
| 20. Researchers blind to ADHD diagnosis/treatment status | Can't tell | Can’t tell | Can't tell | Can't tell |
| 21. Potential confounders defined | Partially | Partially | Yes | Partially |
| 22. Inclusion of potential confounders justified | No | No | No | Partially |
| 23. Methods taken to control confounding | Partially | No | Partially | Yes |
| 24. Large enough sample for statistics | Yes | Can’t tell | Can’t tell | Can’t tell |
| 25. Multiple comparison corrections | No | No | No | No |
| 26. Statistical methods chosen are suitable | Yes | Yes | Yes | Yes |
| 27. Relevant factors included in analysis | Yes | Partially | Partially | Yes |
| 28. Generalizability of the study results discussed | No | Yes | Yes | Partially |
| 29. Declaration of conflict of interest? | Yes | Yes | Yes | Yes |
| 30. Sources of funding identified | No | Yes | Yes | Yes |
| 31. Percentage | 64% | 62% | 66% | 70% |
|  |  |  |  |  |
|  |  |  |  |  |

| **Supplementary Table 1.**  **(continued)** | |  |  |  |
| --- | --- | --- | --- | --- |
|  | Newark et al. (2016) | | Pawaskar et al. (2020) | Turel & Bechara (2016) |
| 1. Background described | Yes | | Yes | Yes |
| 2. Study design reported | Yes | | Yes | Yes |
| 3. Main outcomes described | Yes | | Yes | Partially |
| 4. Aims and objectives reported | Yes | | Yes | Yes |
| 5. Sources and methods of recruitment described | Yes | | Yes | Yes |
| 6. Participant characteristics described | Yes | | Yes | Yes |
| 7. Method of diagnosis based on standardized definition | Yes | | Yes | Yes |
| 8. Sample representative of target population | Partially | | Yes | No |
| 9. Inclusion/exclusion criteria | Yes | | Yes | Yes |
| 10. Excluded/lost to follow-up information reported | No | | Yes | Yes |
| 11. Power calculation used | No | | No | No |
| 12. Comparison between excluded and included participants | No | | No | No |
| 13. Adequate controls | Partially | | Yes | N/A |
| 14. Age/stage specified in relation to diagnosis | Partially | | No | Can't tell |
| 15. Data extraction specified | Yes | | Yes | Yes |
| 16. Variables of interest defined | Yes | | Yes | Partially |
| 17. Reliable measurement of self-esteem | Yes | | Yes | Yes |
| 18. Assessment of ADHD subtype | No | | No | No |
| 19. Error due to recall bias avoided | Can't tell | | Can't tell | No |
| 20. Researchers blind to ADHD diagnosis/treatment status | Can't tell | | Can't tell | Can't tell |
| 21. Potential confounders defined | Partially | | Yes | Yes |
| 22. Inclusion of potential confounders justified | Partially | | Yes | Yes |
| 23. Methods taken to control confounding | No | | Yes | Yes |
| 24. Large enough sample for statistics | Can't tell | | Can’t tell | Yes |
| 25. Multiple comparison corrections | Yes | | Yes | Yes |
| 26. Statistical methods chosen are suitable | Yes | | Yes | Yes |
| 27. Relevant factors included in analysis | Partially | | Yes | Yes |
| 28. Generalizability of the study results discussed | Yes | | Yes | Yes |
| 29. Declaration of conflict of interest? | Yes | | Yes | Yes |
| 30. Sources of funding identified | Yes | | Yes | No |
| 31. Percentage | 63% | | 77% | 69% |
